# Creating an Autonomous Hovercraft for Bathymetric Surveying in Extremely Shallow Water (<1 m)

**DOI:** 10.3390/s23177375

**Published:** 2023-08-24

**Authors:** Meghan L. Troup, Matthew Hatcher, David Barclay

**Affiliations:** 1 Department of Oceanography, Dalhousie University, Halifax, NS B3H 4R2, Canada; 2 Oceans Institute, University of Western Australia, Perth, WA 6009, Australia

**Keywords:** autonomous vehicle, bathymetry, uncrewed surface vehicle, seafloor mapping

## Abstract

Coastal shallow water environments (<5 m) are extremely biodiverse and dynamic yet are often mapped too infrequently or at too low resolutions to capture the important processes occurring in these regions. Common forms of coastal surveying can leave gaps in data in the shallow water zone due to optical instrument capabilities and a vessel’s ability to navigate in this region. One solution to these issues is an autonomous hovercraft that can fly over land and water and begin surveying at sub-meter water depths, bridging the gap between common optical and acoustic surveying methods. The craft’s autonomy is tested via four autonomous flight paths, or missions, and the desired path is compared to both the observed heading and direction of motion. Although the accuracy for each track in the mission varies, most headings and directions of motion of the hovercraft are within 50 degrees of the desired direction. A single-beam echo sounder was used to map the bathymetry of the study site, showing a gently sloping beach.

## 1. Introduction

Nearshore marine environments are some of the most diverse and dynamic areas in the world, making them important areas for mapping studies [1,2]. Coastal areas provide homes and food to not only half the world’s human population, but also a vast number of marine animals including seabirds and marine organisms. These areas are the focus of many underwater mapping endeavors due to the high biodiversity of the nearshore area as well as the need for increased management in the coastal zone [3]. Mayer et al. [4], estimate that less than 18% of the ocean bathymetry has been mapped at a resolution of 1 km. Even though these regions are extremely important ecologically and economically, there are still large gaps in data that large-scale mapping endeavors are consistently aiming to fill [1,2,4,5,6]. Mapping these areas at high resolutions provides detailed geospatial information which can contribute to environmental data, allowing for a more in-depth study of many processes that occur in these dynamic environments. Mapping these areas accurately at high resolutions requires consideration of both the ideal vehicles and instruments to use. In this study, we present an autonomous hovercraft equipped with a single-beam echo sounder as an alternative system for bathymetric mapping in very shallow waters.

In very shallow waters (<5 m), optical methods of mapping are often deployed, including satellite imagery, satellite-derived bathymetry, Lidar, and aerial photography or video [5,6,7,8]. However, these instruments have limited effectiveness depending on the visibility in water and air, and LiDAR and video data sets in particular can be costly to collect and difficult to process [5,8]. Many studies, therefore, combine these aerial optical methods with underwater acoustic data for comprehensive surveys [9,10,11].

In these shallow depths, maneuverability is the most pressing issue. Towed instruments, autonomous underwater vehicles (AUVs), and remotely operated vehicles (ROVs) will eventually reach a depth where they are at risk of damage by the seafloor or other obstacles. Uncrewed surface vehicles (USVs) are platforms that sit on the surface of the water and can maneuver more easily in shallow water regions, depending on their propulsion mechanisms. Recent surveys about the use of these vehicles showed that in the last few decades, studies utilized USVs due to their cost effectiveness, minimized risk to human life, efficiency, and maneuverability in shallow water regions [12,13]. However, even USVs can be limited by depth and geomorphology when the steering or propulsion gear is underwater, such as the system evaluated by Hassan et al. [14], whose propulsion mechanism was 0.4 m below the surface, or the ROAZ II system which has two external motors beneath the water’s surface [15]. USVs can decrease their minimum water depth by decreasing their size, and therefore maximum weight allotment, such as in a study by Giordano et al. [16] using a small catamaran drone (1.35 × 0.85 m), which can be operated in depths of 0–20 m, but cannot run on land as well as water. Similarly, the catamaran USVs developed by Stanghellini et al. [17] and Martorell-Torres et al. [18] use two small underwater propellers which can work in very shallow environments yet cannot be directly deployed from land. Some USVs have adapted their propulsion mechanisms for shallow water by using waterjet or waterjet-inspired propulsion to be able to maneuver in shallow water without risking damage [19]. Even if these propulsion mechanisms do not greatly affect the vehicle’s ability to maneuver in the shallow environment, many surveying instruments are sensitive to underwater noise which could be generated by bubbles and turbulence caused by propellers or water jets [17].

Another solution for the maneuverability problem is an autonomous surface vehicle lacking any underwater steering or propulsion gear, such as a hovercraft, which uses air-side propulsion fans and rudders to maneuver [20]. Hovercrafts are known as air cushion vehicles because they float on top of a thin layer of air. The motor and attached propeller pushes air into the lower part of the hovercraft, enclosed by a tube-like compartment called the skirt. When the skirt fills with air, small holes allow some of the air to escape, creating a small layer of air for the hovercraft to float upon. This layer of air allows the hovercraft to fly with very little friction between the craft and the surface. The frictionless flight as well as the lack of propulsion mechanisms underwater allows the hovercraft to travel over a variety of surfaces including land, ice, and water. Additionally, this low hull drag allows for efficient operation in high-speed currents. While hovercrafts have been used for scientific endeavors before, including sea ice expeditions [21] and terrestrial laser mapping [22], these vehicles have been underutilized when it comes to hydrographic surveying. Ganesan and Esakki [23] developed, modeled, and tested an uncrewed hovercraft created for the purpose of spraying algaecide in remote bodies of water, but this vehicle had a much smaller payload than what would be needed to sustain multiple surveying sensors. Many hovercrafts are constructed by hobbyists for recreational use with off-the-shelf materials, such as the lawnmower engine used in this study. The hobby drone community similarly uses off-the-shelf electronics (i.e., stepper servo motors), relatively affordable on-board computers or autopilots, and open-source software to navigate. When combined into one vehicle, the autonomous hovercraft can be a platform almost entirely created using inexpensive and easily acquired materials.

Studies such as Bachmann et al. [8], Agrafiotis et al. [7], Giordano et al. [16], Bell [24], Stanghellini et al. [17], and Odetti et al. [19] have discussed the difficulties associated with sampling in shallow coastal waters, each using different methods of sampling these regions. The solution presented in this manuscript is an autonomous hovercraft with a single-beam echo sounder and side-scan sonars mounted at the bow. The hovercraft was selected as an ideal vehicle to survey these shallow water environments because of its ability to fly on multiple surfaces, making obstacle avoidance and surveying in shallow areas more reliable. The hovercraft is crafted using light building materials, allowing the vehicle to float in the water, and all electronics are cased in waterproof boxes, making this vehicle particularly low-risk and easy to recover in the event of a motor failure. This is in contrast to many aerial vehicles, which often are not waterproofed and cannot be easily recovered if they fall into the water. The combination of single-beam and side-scan sonars allows for both depth readings and imaging from backscatter, creating a cost-effective system for both bathymetry and seafloor imaging with backscatter. Together, these sonars can be used to map the seafloor bathymetry at high resolutions and also create maps of specific features (such as sediment distribution, bathymetry, and habitat distribution) on the seafloor in these shallow areas. The hovercraft platform with attached sonars provides an inexpensive and efficient system of sampling these dynamic areas that can be applied in any season, allowing for more consistent and high-resolution mapping.

This manuscript will outline the development, field testing, and performance analysis of the SWASH (Shallow Water Autonomous Surveying Hovercraft) system prototype in Section 2, and the results from tests to evaluate the autonomous navigation of the craft and bathymetric surveying capabilities of the sonar instruments in a shallow bay will be presented in Section 3. Section 4 presents our conclusions.

## 2. Materials and Methods

In this section, the development of the SWASH system is discussed, including design, electronics, navigation, software, and sensors used. The methods for field testing this system as well as the following data processing and analysis are also discussed.

### 2.1. Development

#### 2.1.1. Design

**Figure 1:**
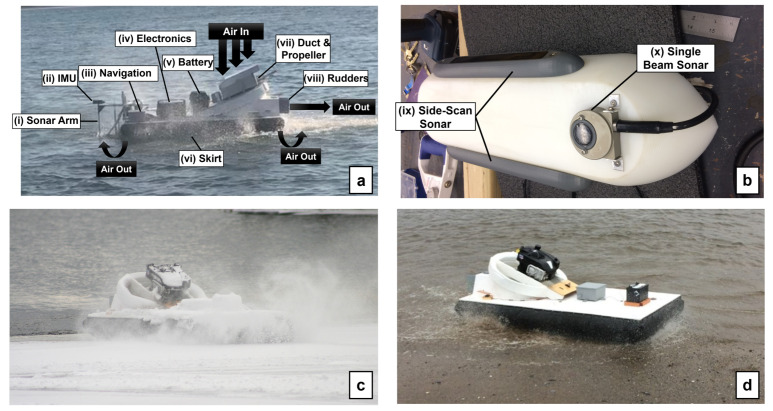
Diagrams of (**a**) the whole hovercraft system and airflow and (**b**) the sonar arm and hovercraft movement between (**c**) water and ice and (**d**) water and land. Labeled features on the hovercraft diagram (**a**) include (i) the sonar arm, shown and annotated in (**b**), (ii) the XSens inertial measurement unit, (iii) the waterproof box containing navigation hardware (i.e., GPS, compass, IMU, autopilot), (iv) the waterproof box containing electronics (i.e., on-board LattePanda computer, power converters, sonar electronics), (v) the 12 V boat battery that powers the electronics, (vi) vinyl tube skirt, (vii) the duct and propeller which create hovercraft thrust and lift, and (viii) twin rudders. The sonar arm (**b**) includes (vi) Imagenex side-scan transducers and (vii) an Imagenex single-beam echo sounder.

**Figure 2:**
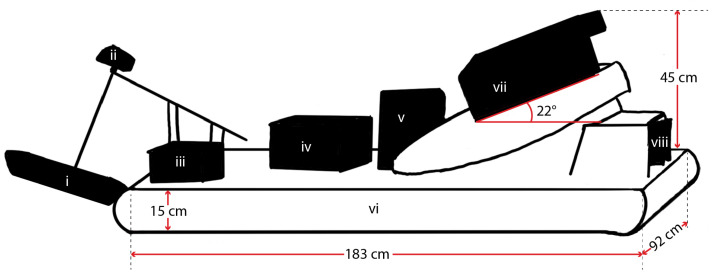
Geometric diagram of the hovercraft, including the numbered features indicated in Figure 1a. The hovercraft’s length, width, and height (subject to the inflation of the skirt) are shown, along with the mounting angle of the motor and positions of the features along the vehicle.

A diagram of the SWASH system’s attributes is shown in Figure 1a. The hovercraft prototype was constructed using plans for the UH-6F Trainer supplied by Universal Hovercraft, in Rockford, IL, USA. The craft has dimensions of 92 by 183 cm and was originally designed to carry a human operator (hovercraft dimensions are shown in Figure 2). The craft’s hull is made of 3.2 mm marine grade plywood fixed to rigid insulation foam, all coated in epoxy resin and fiberglass. It is powered by a single four-stroke gas engine mounted at an angle of 22 degrees from horizontal, with a direct-drive, 24″ thrust propeller connected to the shaft, titled to direct air both down and out the rear. This produces both lift, by forcing air downwards into the skirt through slots in the craft’s frame, and thrust, by forcing air through a rear-facing exhaust. Because the propulsion mechanisms are not situated below the vehicle, the hovercraft can transition seamlessly between surfaces, such as land, water, and ice (Figure 1c,d). The hovercraft has a vinyl bag skirt with two drainage holes in the aft for any water that accumulates in the skirt during flight. Twin, 3D-printed rudders are mounted in the rear exhaust to provide steerage. The engine and air intake system are protected from salt-water spray by an aluminum case, while an elongated pipe directs engine exhaust away from the craft.)

An aluminum outrigger arm capable of raising and lowering a hydrodynamic-shaped outrigger hull is mounted to the front of the craft (Figure 1b). The outrigger is made from high-density polyethylene and carries the single beam and dual side-scan transducers at a depth of 9 cm below the water’s surface when lowered).

A vented box fixed to the topside of the hull contains a lead-acid 12 V marine battery that powers all electronics on the craft. Two waterproof boxes contain electronic hardware for power management, steering and throttle control, navigation, sonar operation, and data acquisition and storage. The boxes are positioned such that the craft’s center of mass is located on the propeller fan’s center of lift, thus balancing the hull so it maintains horizontal pitch and bank attitudes while in motion.

Three servo motors interface the control electronics to the craft’s engine and steering system. One controls the twin rudders through a yoked steering system, one directly drives the engine’s throttle, and the third is connected to an engine kill switch. A linear actuator is used to lower and raise the outrigger.

#### 2.1.2. Electronics and Navigation

**Figure 3:**
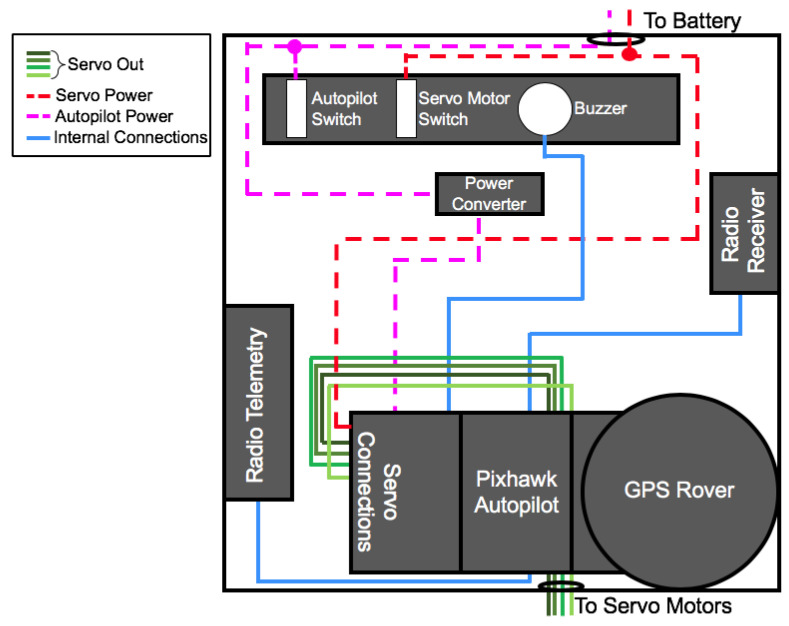
Diagram including the contents and layout of the waterproof box containing navigation equipment (i.e., (iii) from Figure 1a). Dashed lines indicate power connections, blue lines indicate wiring between the navigation equipment, and green lines indicate the outputs from the autopilot to servo motors controlling the throttle and steering, the linear actuator, and a switch to control autonomous hovercraft modes.

**Figure 4:**
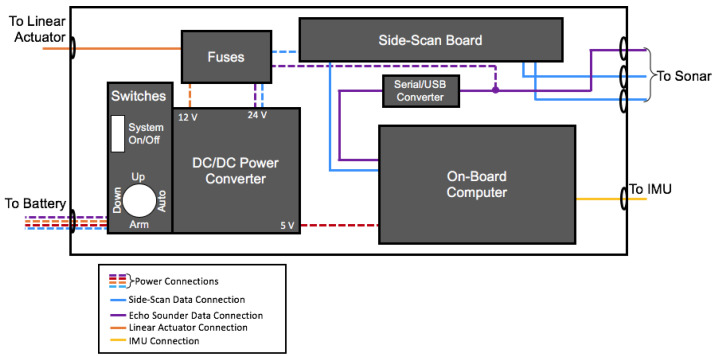
Diagram including the contents and layout of the waterproof box containing electronics for the hovercraft (i.e., (iv) from Figure 1a). Dashed lines indicate power connections, purple lines indicate echo-sounder inputs and power, blue lines indicate side-scan sonar connections, yellow lines indicate XSens IMU inputs and power, and orange lines indicate connections for the linear actuator.

Electronic hardware is split up into two waterproof boxes: one containing the navigation electronics, and another containing the computing boards, power management, and onboard computers (detailed layouts of these boxes are shown in Figure 3 and Figure 4). The electronics box (labeled iv in Figure 1) contains a power distribution block, transforming the 12 V supply to three protected circuits of 5, 12, and 24 V by a series of DC-DC converters. A LattePanda single-board embedded system running custom and manufacturer-provided software for the control and data acquisition of the single beam and side-scan sonar is connected to the transducer breakout board through a serial interface. The details of the instruments and electronics deployed on the SWASH system are described in Table 1.

The box containing navigation sensors includes a 10 degrees-of-freedom inertial measurement unit (IMU), Global Positioning System (GPS) receiver, autopilot computer, two radio telemetry antennas, and servo motor connections. This box is placed at the bow of the craft to minimize the impact of the disturbance of the magnetic field caused by the engine’s magneto on the magnetometers in the IMU. Additionally, the navigation electronics are isolated from shock and vibrations using compliant mounts to improve IMU performance. The IMU contains 3-axis accelerometers, 3-axis gyroscopes, and a barometer that feed their data, along with the GPS output, to the navigation computer, the Pixhawk 2.1. The Here+ RTK GPS has Real Time Kinematic (RTK) capabilities and can determine the hovercraft’s instantaneous position with a maximum positioning accuracy of 2.5 cm both horizontally and vertically. This RTK GPS takes several hours to reach these centimeter-sized resolutions; therefore, during the survey analyzed in this chapter, the GPS was set to a larger desired positioning accuracy to decrease the amount of time to set up the base station. The autopilot and attached sensors (autopilot IMU, compass, and GPS) collect data at a sample rate of approximately 25 Hz.

A second IMU (XSens MTi-20) is mounted at the top of the arm used to raise and lower the sonar outrigger. It collects the local accelerometer, magnetometer, and gyrometer data to determine the precise location and orientation of the sonar transducers. The sample rate for this IMU is 2000 Hz.

When the craft is set to autopilot along a pre-programmed mission, the computer controls the servos and linear actuator, and thus the flight and operation of the hovercraft. An antenna and transceiver (mRo SiK, 915 MHz) provide a connection between a base station and the autopilot, providing real-time system updates to shore, and allowing re-programming of the mission in certain flight modes. A conventional 75 MHz band receiver allows for remote-controlled flight, which is able to supersede the autopilot at any moment.

#### 2.1.3. Acoustic Sensors

The outrigger hull is mounted with multi-frequency (260, 330, or 800 kHz) Imagenex Yellowfin side-scan sonar transducers with a beam width of 30 degrees at the highest frequency and an Imagenex 852 single beam echo sounder (675 kHz) with a beam width of 10 degrees. The pulse length of the single beam echo sounder is 100 microseconds in 10 μs increments. For the initial testing, the side-scan soar was run at 800 kHz only and had a ping interval of 53 ms. The resolution for these sensors depends on the range setting of the transducers. For this study, the side-scan transducers were set at a range of 10 m horizontally, while the single-beam sonar was set to a vertical range of 5 m. Using these settings, the resolution for both sonar instruments is 0.01 m. Before field testing, both side-scan and single-beam sonars were tested in the Dalhousie Aquatron facility in the Pool Tank (dimensions 15.24 m in diameter, 3.54 m depth at perimeter, and 3.91 m depth in the center) to ensure the sensors were working correctly and test that the backscatter from the tank floor was at an accurate range.

#### 2.1.4. Software

Data collection for the system occurs on two platforms: the on-board computer logs sonar acoustic data and transducer motion data from the XSens IMU mounted on the outrigger arm and the navigation computer logs GPS, IMU, telemetry, and metadata having to do with the autopilot function. Data from both sonar instruments can be collected automatically or manually. Automatically, single-beam echo sounder data is collected from the port using a custom Python script. Side-scan data is collected automatically using a modified C++ executable provided by the manufacturer. A batch script allows these two codes to be initialized before deployment. Data from the XSens IMU is collected using the manufacturer’s software.

Data collected by the autopilot can be assessed in real-time via a telemetry link or analyzed after the deployment using the open-source software, Mission Planner, developed by Michael Oborne for the open-source Ardupilot Mega autopilot project [25]. While a variety of data is collected during deployment to assess the health of the compass, the ability of data logging, the stability of the Extended Kalman Filter (EKF), the vibrations of the autopilot, the GPS fix, and the strength of the connection between the computer and hovercraft telemetry radios, the user can choose which data is recorded. During the development process, Mission Planner was used to optimize steering, speed, and navigation by adjusting limits and coefficients used in the control algorithms for the throttle and rudder servos. This program uses magnetometer, accelerometer, and gyrometer data to calculate roll, pitch, and yaw via EKF and can also use this filter to predict and adjust these values to control autonomous servo outputs for the steering and the throttle.

### 2.2. Field Testing

During the development phase of the SWASH system, several field tests were carried out to examine the hovercraft’s flight on many surfaces. Land tests were carried out at Wickwire Field on the Dalhousie University Studley Campus. Ice and snow tests were carried out at Williams Lake, Nova Scotia (Figure 1c), and water and land tests were carried out at Horseshoe Island Park, Halifax, Nova Scotia (Figure 1d). Before each field test commenced, the hovercraft system was set up and initialized based on the specific field site and goals for the test. A base station, including a laptop computer attached to a telemetry antenna and Here+ RTK GPS base station antenna, was set up at the same location for each field test. The autopilot was turned on and initialized along with the GPS, which must localize using a number of satellites. Once the GPS reaches the desired level of accuracy, field tests began, focusing on programming, calibrating, and tuning the hovercraft for autonomous flight. Flight plans were created using the software Mission Planner, considering the approximate wind direction and whether there was any wave action in the area. All sensors were turned on and data recording was initialized. The hovercraft was then driven to face the first waypoint and switched into autonomous mode. During the autonomous flight, observations about the hovercraft flight were made, and once the flight was completed and the hovercraft returned to land, these observations are used for tuning parameters. Flight mode can be switched at any time to a different mode including manual, in order to take over control of the hovercraft, or hold, which changes all hovercraft functions to zero, using the transceiver.

#### 2.2.1. Study Site

**Figure 5:**
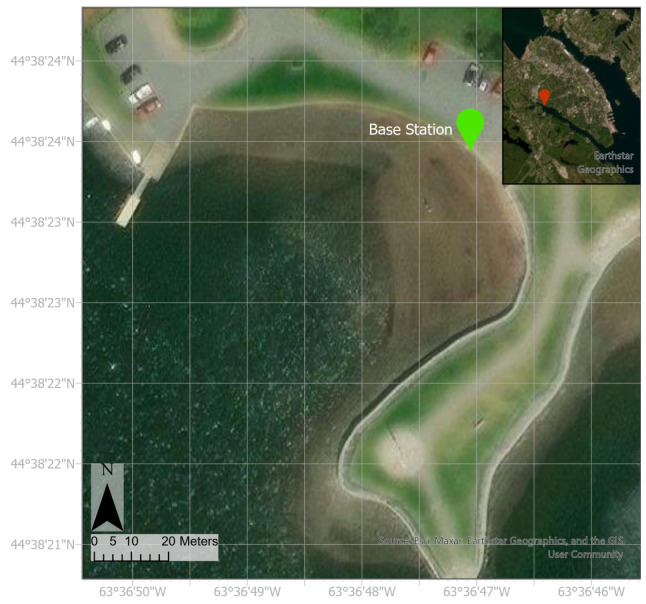
Satellite image of Horseshoe Island Park. A red pin on the Halifax Peninsula indicates the location of Horseshoe Island Park within the city. A green pin indicates the location of the base station for the surveys discussed in this manuscript.

The SWASH system’s autonomous performance was field tested at Horseshoe Island Park beach in Halifax, Nova Scotia (−63.61° W, 44.64° N), shown in Figure 5. Horseshoe Island Park is a small beach along the Northwest Arm that has a gently sloped topography. This beach is located at the inland end of the Northwest Arm inlet and is further sheltered from waves by a small peninsula of higher elevation reaching into the arm that protects the beach. A seawall lines the beach and the water reaches this seawall at high tide. The maximum depth in this region of the Northwest Arm is approximately 6 m. Tides in this area are semidiurnal.

The field test analyzed in this study was performed on 28 April 2019, from approximately 9:30 to 11:30 a.m. Low tide occurred at 10:30 a.m. According to hourly data from the two closest weather stations: one at Windsor Park (−63.61° W, 44.66° N) and one at the Halifax Dockyards (−63.58° W, 44.66° N), the wind speed increased throughout the morning, and wind direction fluctuated by one or two degrees each hour. The base station was set up on top of the seawall. The accuracy for the Here+ RTK GPS was set to 0.7 m for the duration of data collection during this field test to minimize the time needed for field set-up.

#### 2.2.2. Tuning Autopilot Parameters

In order to improve the craft’s ability to navigate along the preprogrammed path, certain variables within the autopilot that alter the vehicle’s speed, throttle, steering, and navigation must be tuned. The variables changed during tuning affect the steering and throttle servo motors’ maximum, minimum, and cruising default positions. These variables can also affect the vehicle’s motion and speed as it approaches a programmed waypoint. The website Ardupilot was used as a reference for using Mission Planner for autopilot setup and is useful for tuning parameters especially [25]. The tuning process consists of changing Proportional, Integral, and Derivative (PID) controller parameters, which will allow the vehicle to adapt quickly to errors in the autonomous path. PID controllers are the most commonly used controllers in the field of robotics because they are cost-effective, simple, and effective [26]. Unfortunately, as feedback controllers, they can only adapt a path and respond to errors once they have been recorded [27]. For autonomous applications with mostly straight paths, PID controllers are often sufficient. However, for applications that include turns or for vehicles with unique movement and control mechanisms, a different controller is necessary. Feedforward (FF) controllers use a planned path and a set “lookahead” point to try to predict where errors may spring up. These two controllers working in tandem can decrease errors and prevent large instabilities. Though significant guidance for configuring the steering systems of propeller-driven autonomous surface vehicles and autonomous aircraft, the hovercraft has unique attributes such as a sole thrust and steering motor and no hull friction (including lateral resistance), requiring the unique tuning of autopilot parameters.

The SWASH system has one propulsion motor, and therefore one mechanism controlling both speed and throttle. In addition to managing maximum, minimum, and cruising speeds, this tuning process can also be used to change FF, P, I, and D gains [25]. The P gain controls the short-term consistency of vehicle speed. If the P gain is too high for a specific vehicle, the speed will change sporadically. If this variable is set too low, the vehicle will take too long to reach cruising speed. The I gain controls long-term consistency of vehicle speed. The I gain is too large if the vehicle speed is constantly either too low or too high and the vehicle does not reach the cruising speed. The D gain stabilizes the vehicle speed.

The steering is tuned using the FF, P, I, and D gain parameters as well. The minimum and maximum turn rates for the vehicle can be set. The FF gain affects the turn rate of the steering (or motor-controlling steering), which controls the speed of the hovercraft as it turns and combines throttle and rudder outputs. The larger the FF, the faster the turn rate. The P gain controls the steering on short-term time scales. If FF is set correctly, and thus predicts the potential steering errors, the P gain values do not need to be adjusted. I gain controls the turning in the long-term turn rates, and the D gain stabilizes.

The navigation can be tuned using the L1 controller, which is a high-level steering controller that accepts latitude and longitude points, then outputs a lateral acceleration. The lateral acceleration is then used when controlling lower-level controllers such as the steering rate. The lateral acceleration control period can be changed to decrease weaving along straight pathways and increase the sharpness of turns, while the lateral acceleration control damping can be changed to improve vehicle control when paths contain many turns and waypoints are close together.

After tuning these parameters on 28 April, four autonomous flight plans were completed during this field test, three of which were performed with sonar instruments deployed in the water. These four flight plans were used to analyze the accuracy of hovercraft flight and demonstrate the precision and accuracy of bathymetric mapping on the platform.

### 2.3. Post-Processing and Data Analysis

All data were georeferenced and synced to GPS time from the RTK GPS using the two IMU accelerometer data sets. The autopilot IMU data were collected on GPS time intervals while the XSens IMU were collected using the onboard computer, which can have time differences. The GPS time is calculated from GPS week and elapsed seconds since that week began. The two vertical accelerometer time series were cross-correlated and the lag at the maximum peak was used to alter the onboard computer time to match the GPS time.

The hovercraft path accuracy was evaluated using both the observed heading computed by the GPS and the direction of motion. The direction of motion was computed by finding the direction traveled from one GPS coordinate to the next. The accuracy of the hovercraft was determined by wrapping all angles between the range of [−180, 180] before using Equation (1) to compute a difference between desired heading ($\theta_{d}$) and observed heading ($\theta_{o}$). The desired heading was computed by taking the direction in a straight line from the hovercraft’s instantaneous position to the following waypoint. The metric, $\Delta_{H}$, given by

(1) $$\Delta_{H}=\left|\theta_{d}-\theta_{o}\right|,$$

provides a measure of the performance of the hovercraft’s steering controller tuning and directly evaluates the ability of the craft to point its bow at the next waypoint.

Path accuracy between the desired heading and observed direction of motion ($\theta_{m}$) is computed in Equation (2). The metric, $\Delta_{M}$, given by

(2) $$\Delta_{M}=\left|\theta_{d}-\theta_{m}\right|,$$

is an evaluation of the craft’s ability to navigate toward the next waypoint and correct for errors. The first accuracy metric, $\Delta_{H}$, is calculated for every observed heading, and the second accuracy metric, $\Delta_{M}$, is calculated each time the instantaneous hovercraft position changes.

The single-beam echo sounder transducer depth and horizontal position data were corrected to account for the pitch, roll, and yaw of the vehicle using quaternion rotations. Quaternions rotate rigid bodies along the shortest path from one orientation to another and are often used in place of rotation matrices because they do not require a specific order of rotation [28]. The quaternions are calculated automatically on board the XSens sensor.

**Figure 6:**
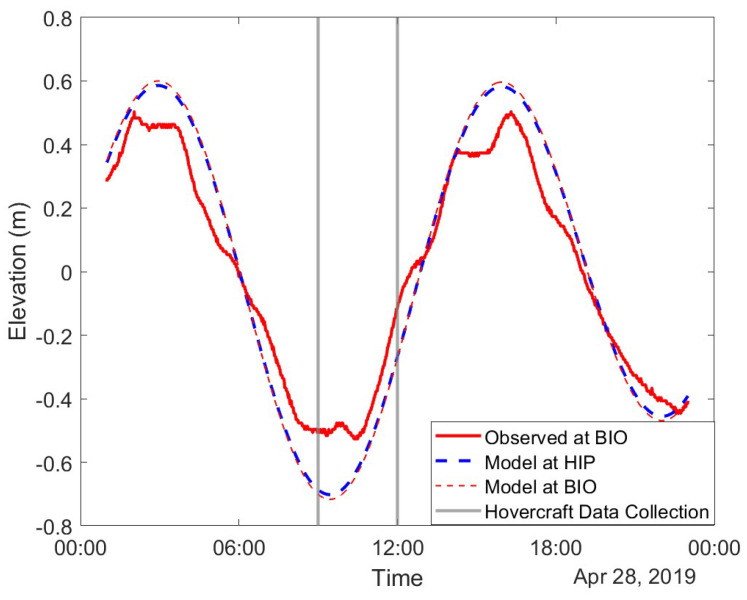
Observed water level data at a buoy near the Bedford Institute of Oceanography (BIO) at position −63.62° W and 44.68° N and compared to modeled elevation data at the buoy’s position and at Horseshoe Island Park (HIP) using the WebTide model. Gray vertical lines indicate the period during which the hovercraft was used. Red tide elevations indicate BIO buoy positions and blue indicates the modeled data for HIP. The modeled data are very similar between the two positions, though they are separated by the Halifax peninsula. While the modeled tidal elevation data are very similar, the observed elevation data have a smaller range and are slightly out of phase from the modeled BIO elevation data.

After corrections for pitch and yaw were made, the depth data were corrected for changes in water level over the total survey time. Water level data sampled at one-minute intervals from a bouy at the Bedford Institute of Oceanography station at −63.62° W and 44.68° N were used for this correction. Corrections were performed by locating the shallowest water level datum during the hovercraft sampling period and the echo sounder depth datum that corresponded during that time. The change in water level between the minimum and each previous or subsequent water level was subtracted from the echo sounder depths at each matching time stamp. Observed tides for the Bedford Institute of Oceanography station and modeled tides at this location and at Horseshoe Island park are shown in Figure 6.

The approximate range of single-beam echo sounder footprint radius on the seafloor is between 1.75 cm at 0.2 m depth and 35 cm at 5 m depth. Although the recommended minimum detectable depth for this instrument is 0.5 m, the echo sounder can be utilized in shallower depths, and these surveys aimed to test the limits of the hovercraft and these instruments to determine how effective these surveys can be. The single-beam echo sounder provides a return from a single point-location from the location with the fastest travel time. If the echo sounder footprint is large, this can add uncertainty to the georeferenced depth. Since the water in the area sampled with this echo sounder does not exceed 5 m, we can assume the uncertainties are quite small due to the small footprint size of the echo sounder beam. The sonar data was processed using MATLAB to produce maps of the seafloor depth data corrected for vehicle motion. Land positions and low-tide lines were collected using georeferenced satellite imagery. The hovercraft-derived depths were compared to bathymetry provided by the Canadian Hydrographic Service [29]. The CHS depth data were collected using a combination of multibeam sonar and LiDAR data collected over a period of years, 1998–2005 and 2014, respectively. These data sets were combined by CHS using the shallowest depth for any overlapping coordinates, discarding the deeper data points. The CHS depth data within 1 m of hovercraft positions were subtracted from hovercraft-derived depth data. Mean absolute error and root mean squared error values were calculated as well to compare the depth data sets.

## 3. Results

The results of hovercraft development are outlined in this section. First, autonomy is evaluated based on how accurate the vehicle’s path is when compared to a pre-programmed mission. The results of the depth from single-beam sonar are then compared to previously collected data to evaluate the accuracy of bathymetry.

### 3.1. Autonomy

Four autonomous hovercraft missions were performed on 28 April 2019. The cruise speed during this survey date was set to 1 m/s and varied between a range of 0.5–1.5 m/s for all data collected. Each mission took between approximately 3–6 min to complete and all were carried out from 10–10:42 a.m. Table 2 shows the duration of each mission and the number of waypoints planned.

**Figure 7:**
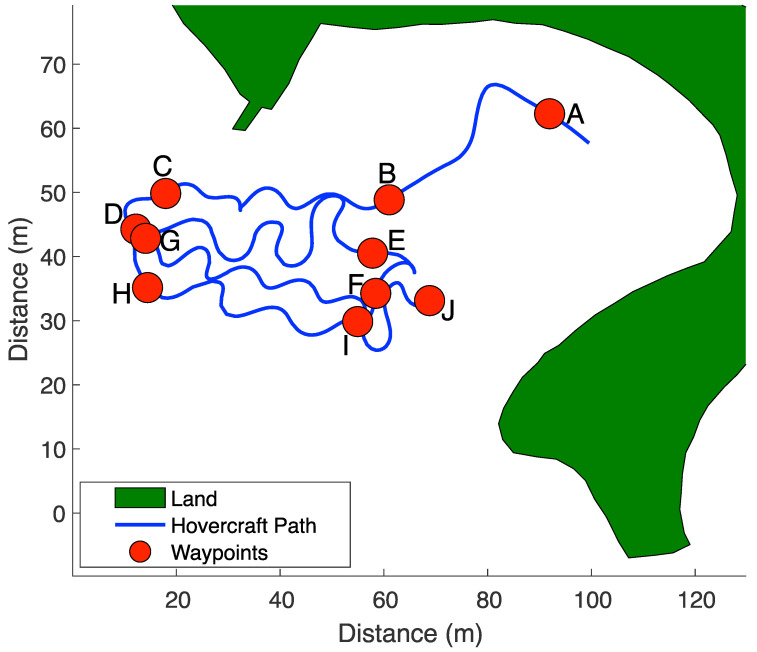
Map of hovercraft Mission 2. The hovercraft path is shown in blue. Planned waypoints are labeled by letter and shown by a red dot. The land is shaded in green.

The hovercraft’s autonomous path was tested for accuracy by comparing the hovercraft’s desired heading (straight line to waypoint) to the observed heading and by comparing desired heading with the direction of motion, due to the propensity of the hovercraft to drift (i.e., small $\Delta_{M}$, large $\Delta_{H}$). Longer hovercraft tracks (A–B, B–C, D–E, F–G, H–I, I–J of Mission 2, shown in Figure 7) are used for evaluating the accuracy of hovercraft autonomy. The results from Mission 2 are fully presented in this manuscript because it was the first full mission completed with the sonar instruments deployed. Similar analyses were carried out on all missions in the process of control system tuning.

**Figure 8:**
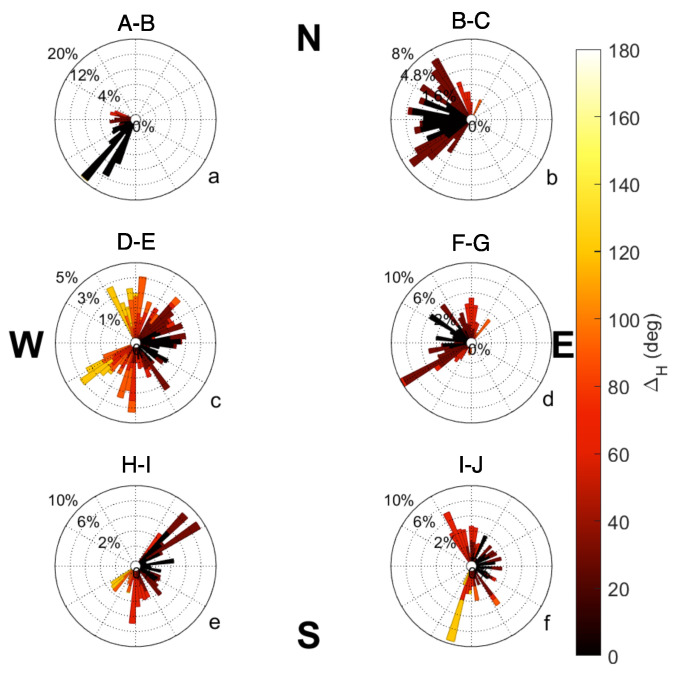
Differencesin headings for each long track in Mission 2. The individual subplots indicate paths (**a**) A–B, (**b**) B–C, (**c**) D–E, (**d**) F–G, (**e**) H–I, (**f**) I–J. Directions indicate the observed headings and vector length is a factor of heading frequency. The color indicates the difference between the observed ($\theta_{o}$) and the desired heading ($\theta_{d}$).

The completed Mission 2 track is shown in Figure 7. Waypoints were assigned letters in order to identify flight path segments. Polar histogram plots comparing the desired heading (pointing directly to the next waypoint) versus the observed heading are shown in Figure 8. Bars show the direction of the observed heading, and the inner dashed circles represent the frequency of headings. The bin width for each bar is 10 degrees. Darker reds indicate that the observed heading for each track was closer to the desired heading. Some plots show a wider range of low differences because the desired heading changes as the position of the hovercraft changes.

The selected flight plan had a total of 10 tracks, 6 of which will be discussed in this manuscript. Headings for tracks A–B (Figure 8a) are uniform as well as accurate, with most observed headings pointing in the south/southwest direction and falling within 20 degrees of the desired heading. Other tracks for this mission, including B–C, D–E, F–G, H–I, and I–J (Figure 8b–f) exhibit higher variability in observed headings. Tracks D–E and I–J (Figure 8c,f) in particular have a higher percentage of large $\Delta_{H}$ values.

**Figure 9:**
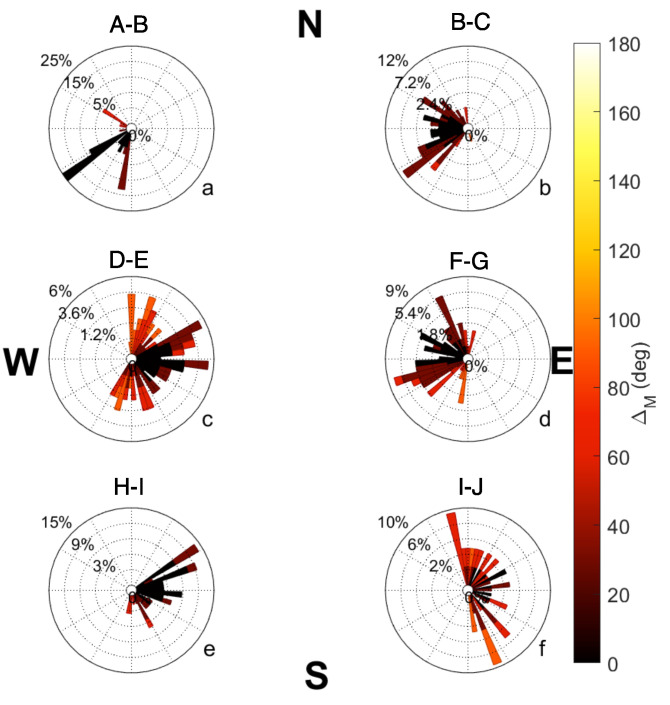
Differences in direction of motion and the desired heading for each long track in Mission 2. The individual subplots indicate paths (**a**) A–B, (**b**) B–C, (**c**) D–E, (**d**) F–G, (**e**) H–I, (**f**) I–J. Directions indicate the observed headings and vector length is a factor of heading frequency. The color indicates the difference between the direction of motion ($\theta_{m}$) and the desired heading ($\theta_{d}$).

The $\Delta_{M}$ values were evaluated for the six tracks from Mission 2 in Figure 9. While higher variability in the direction of motion is shown in tracks B–C, D–E, F–G, and I–J (Figure 9b–d,f) similar to Figure 8, the variability in the direction of motion is less than the variability of observed headings. While differences of 120 to 140 degrees are shown in Figure 8c,e,f, the maximum differences in Figure 9 are approximately 100 degrees. The increases in $\Delta_{M}$ and $\Delta_{H}$ do not consistently occur at similar points between missions. The direction of motion shows a more accurate path than the observed headings for this mission.

**Figure 10:**
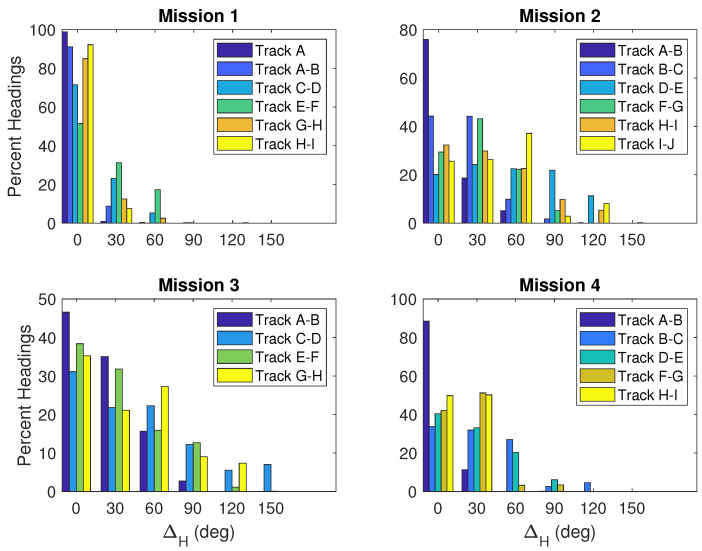
Histograms showing differences in the desired heading ($\theta_{d}$) and observed heading ($\theta_{o}$) for non-turning tracks for each mission completed on 28 April 2019.

**Figure 11:**
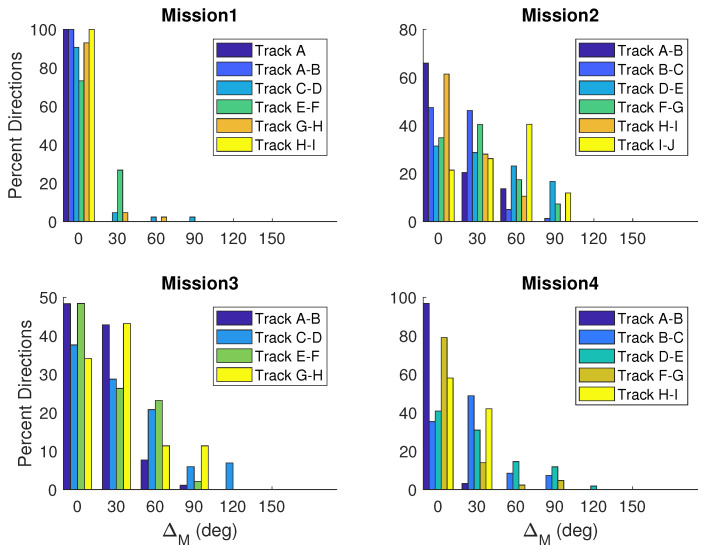
Histograms showing differences in the desired heading ($\theta_{d}$) and direction of motion ($\theta_{m}$) for non-turning tracks for each mission completed on 28 April 2019.

The delta values are compiled for all four missions completed on 28 April 2019, in Figure 10 and Figure 11. Differences are binned in 30-degree groups and the percent of headings per track are computed. Figure 10 shows $\Delta_{H}$ values. Mission 1 is the most accurate with all differences less than 90 degrees. Missions 2 and 3 both have $\Delta_{H}$ values exceeding 120 degrees, and Mission 3 has the largest $\Delta_{H}$ values of 150 degrees. When $\Delta_{M}$ is evaluated in Figure 11, the $\Delta_{M}$ and $\Delta_{H}$ values differ for each mission, but the trends are similar between these two methods. Missions 1 and 4 both show a higher percentage of differences close to zero. Tracks in Mission 3 also show a higher percentage of small differences both centered around 0 and 30 degrees.

### 3.2. Bathymetry of Horseshoe Island Park

During an average hovercraft flight, the pitch and roll (two rotations that affect perceived echo-sounder depth the most) rarely reach above half the conical beam width of the echo sounder (5 degrees); thus, corrections on ping-by-ping measurements are very small. The majority of these corrections come from adding the measured depth of the echo-sounder below the water’s surface onto raw echo-sounder depth, unlike a typical survey boat or towed vehicle which will have significant heave, pitch, roll, and other motion corrections.

**Figure 12:**
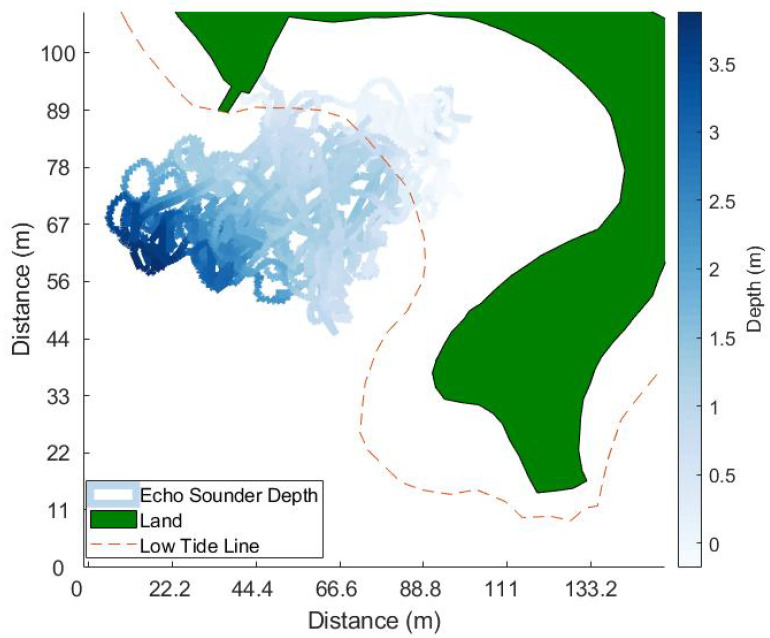
Map of Horseshoe Island Park with bathymetry collected with the hovercraft’s single-beam echo sounder. The land is filled in with green, the low tide line is indicated by the red, dashed line.

**Figure 13:**
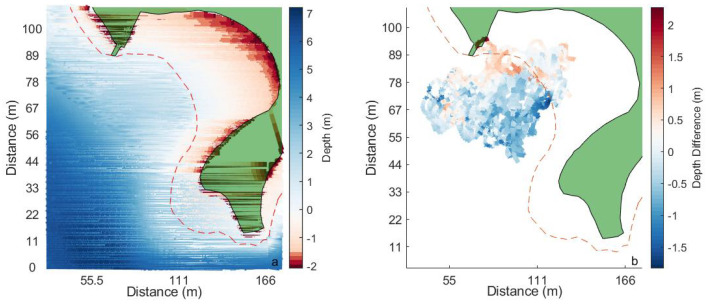
Bathymetry from multibeam and LiDAR data (**a**) from the Canadian Hydrographic Society in the Northwest Arm, Halifax. CHS bathymetry is subtracted from Hovercraft bathymetry in (**b**). The low-tide line is indicated with the red, dashed line. Land is shaded in green.

Echo-sounder depths over the hovercraft’s entire flight path are shown in Figure 12. The land is green and the low-tide line is shown by a red, dashed line. At high tide, the water can reach the seawall (land boundary). Single-beam echo sounder data from the entire surveying period are shown, regardless of manual or automatic mode. The bathymetry in this area is shallow and the echo sounder was able to map the gently sloping beach. In Figure 13b, the bathymetry from the single-beam sonar is compared to existing combined multibeam sonar and LiDAR depth data provided by the Canadian Hydrographic Society (CHS). These data show a gently sloping beach, which supports visual observations made at the study site. When the CHS data is subtracted from the single-beam depth data, the differences are more apparent. Positive values indicate that either the hovercraft data were greater (i.e., more positive) or that the CHS data were much more negative (i.e., LiDAR measurements on land), while negative differences suggest that CHS data were greater than hovercraft-derived depths. Larger differences can be seen around the edges of the single-beam sonar data path and especially along the low-tide line. The largest difference can be seen in the top left-hand corner of the data, where differences were greater than 2 m. Mean absolute error between the two depth data sets was 0.42 m, and root mean square error was 0.53 m.

## 4. Discussion

While the GPS used in this study has RTK capabilities, the positional accuracy was set to 0.7 m rather than the more common centimeter-range for most studies using RTK. Regardless, this study still uses a differential positioning method, using the base station to correct the rover’s GPS position (i.e., GPS deployed on the SWASH system) [30,31]. This decreases the positional error in the rover GPS, as well as decreases or eliminates certain sources of error, such as clock drift, satellite orbit shift, and ionospheric or tropospheric delays. This reduction in error sources will reduce noise in the error of the GPS, even with a positional accuracy of larger than centimeter resolution [31,32].

The hovercraft is able to reach the planned waypoints for every mission. In every case, when $\Delta_{H}$ values are compared with $\Delta_{M}$ values, the latter is less variable and more accurate, creating a more accurate metric for analyzing hovercraft performance. Figure 10 and Figure 11 both indicate that Missions 1 and 4 are more accurate than Missions 2 and 3, due to a higher proportion of delta values between 0 and 60 (and thus a lower proportion of delta values between 60 and 180). The sonar arm was deployed for Missions 2–4, suggesting that the accuracy of Mission 1 is due to the lack of drag generated by instruments in the water. Hovercraft flight can be affected by factors external to the system such as wind speed and direction, wave action, current speed, and surface slope, or internal factors such as skirt inflation, parameter tuning, and engine performance. Any combination of these factors could cause hovercraft flight accuracy to decline.

Wind data from 28 April 2019 collected from the Halifax peninsula show the wind speed increasing throughout the field test, indicating that some of these external factors were present and could have affected the accuracy of the hovercraft. Wind direction from the Halifax Dockyards and Windsor Park changed by 10 degrees over the hour during which data was collected, although, based on observations during surveying, the wind direction changed much more frequently. If the hovercraft path was parallel to the direction of the wind, the accuracy of the direction of motion was likely to be higher, however, if the wind direction was perpendicular to the hovercraft path, the accuracy decreases as the hovercraft can be blown off track easily due to the near-friction-less motion against the water or ground surface. During the missions in which the sonar arm was in the water, water speed may have also had an effect on the accuracy of the hovercraft, as the tide switched between ebb and flood during these autonomous surveys. Regardless of the differences in accuracy between autonomous missions, they were all deemed successful because they managed to reach programmed waypoints without going too far off track.

The differences between hovercraft-derived depths and the bathymetry collected via Lidar and multibeam sonar by the Canadian Hydrographic Society (CHS) vary between 0–2 m in some regions. These differences are larger than what is acceptable in terms of the International Hydrographic Organization’s (IHO) standards [33]. Furthermore, the mean error and root mean squared error between the hovercraft-derived depths and depth data from CHS are very similar, indicating that the sources of error all contribute on the same order. While hovercraft motion can affect the depth data minutely, the hovercraft is quite stable, with pitch and roll measurements rarely exceeding 5deg. Thus, these error sources are more likely due to the positional accuracy of the hovercraft or external forces. Some differences in depth could be due to sediment transport changing the local geomorphology over the years and the water level data used for tidal corrections being inaccurate for this area. The closest water level station to the study site is in a different body of water and local differences in tide phase and current speeds could affect the accuracy of tide corrections. Figure 6 shows the water level data at the Bedford Institute of Oceanography, which indicates a total elevation change of approximately 0.5 m during the hovercraft surveys. Further surveys must be carried out to validate the sources of error between the SWASH and CHS data sets and isolate which sources of error can be reduced or eliminated through post-processing.

## 5. Conclusions

The autonomous hovercraft represents a low-cost, low-risk surveying platform that can be deployed for high-resolution benthic monitoring and bathymetry mapping projects in relatively small areas. While there are significant challenges that arise when creating an autonomous craft, including the vehicle’s proclivity to drift, and the power being split between lift and thrust as a consequence of a single, tilted gas engine, the autonomy of the platform has been ultimately successful. The hovercraft system development was deemed successful because the vehicle could complete a mission with both the sonar instruments in and out of the water. Though accuracy can vary from mission to mission and track to track within missions, collecting these statistics can infer important information about what parameters need to be altered to increase the accuracy of these autonomous missions. Consideration must be made with respect to the types of sensors deployed in the water due to hovercraft movement. The observed drifting does not affect the accuracy of downward-pointing instruments such as the single-beam sonar, nor would it affect non-imaging instruments such as those surveying conductivity, temperature, and depth (CTD), current meters, or other instruments of these types. While the depth data derived from the SWASH platform appeared accurate, showing a gently sloping beach where the slope increases past the low-tide line, these data were significantly different than existing bathymetric measurements from CHS in some locations. Further analysis must be carried out to compare SWASH-derived depth data with updated bathymetric measurements and localized water level data to determine the cause of these differences.

When collecting bathymetry with a single beam echo sounder, the hovercraft’s difficulty in following straight tracks is not an issue, and can actually provide data with high spatial resolution and coverage at a study site when data from several autonomous missions are combined. However, when mapping using side-scan imagery, a straight track is necessary to minimize motion artifacts and improve the quality of mosaics. While no quantitative analysis on hovercraft pilot performance was carried out, observations made during field testing show that the hovercraft’s variation in the heading is easier to correct manually than autonomously. For future surveys in which side-scan sonar is deployed on the SWASH system, the hovercraft should be piloted manually to decrease changes in the along-track heading.

Future work with the SWASH system development will include physical changes with respect to the SWASH prototype that may help the hovercraft perform better during autonomous missions (i.e., two motors instead of one, changes to the steering mechanisms. electric motors). Other upgrades will be made with respect to the power source, electronics, and waterproof housings. During future experiments, the RTK GPS capabilities should be further exploited (i.e., localized to higher spatial resolutions) to increase the possible resolution of the resulting data. The SWASH system was originally built to be a mapping platform; however, the vehicle has the capability to carry and deploy more instruments and will be upgraded to accommodate a variety of sensors. Thus, future prototypes will be built with multi-sensor integration in mind.

## Figures and Tables

**Table 1:** Instruments deployed on the SWASH system with their specifications, including manufacturer and model and measurements made.

Instrument	Manufacturer/Model	Measurement
Echosounder	Imagenex 852 Port Coquitlam, BC, Canada	Profile backscatter at 675 kHz
Side-scan Sonar	Imagenex Yellowfin Port Coquitlam, BC, Canada	Swath backscatter at 800 kHz
Inertial Measurement Unit	XSens MTI-20 Henderson, NV, USA	XYZ acceleration, orientation, quaternion
Autopilot	ProfiCNC HEX Pixhawk 2.1 Xiamen, China	XYZ acceleration, orientation, autonomous controls
GPS	ProfiCNC HEX Here+ RTK GPS Base and Rover Xiamen, China	Instantaneous vehicle position, ground speed
Telemetry Radio	mRobotics mRo SiK Telemetry Radio 915 mHz Chula Vista, CA, USA	Communication between base and rover
On-board Computer	LattePanda LattePanda V1 Shanghai, China	Sonar and IMU data collection

**Table 2:** Autonomous test missions carried out on 28 April 2019, duration of the missions in minutes, and the number of waypoints planned as the missions.

Mission	Duration (min)	Waypoints
1	3:02	9
2	5:50	10
3	4:20	7
4	4:13	9

## Data Availability

Not applicable.

## Abbreviations

The following abbreviations are used in this manuscript:

AUV	Autonomous Underwater Vehicle
CHS	Canadian Hydrographic Service
CTD	Conductivity, temperature, depth
D	Derivative
FF	Feed Forward
GPS	Global Positioning System
I	Integral
IMU	Inertial Measurement Unit
LiDAR	Light Detection and Ranging
P	Proportional
RTK	Real Time Kinematic
ROV	Remotely Operated Vehicle
SAS	Synthetic Aperture Sonar
SONAR	Sound Navigation and Ranging
SWASH	Shallow Water Autonomous Surveying Hovercraft
UAV	Uncrewed Aerial Vehicle
USV	Uncrewed Surface Vehicles
